# Prurigo Pigmentosa Following Bariatric Surgery: A Comprehensive Clinicopathological Review

**DOI:** 10.1155/drp/1993385

**Published:** 2026-01-09

**Authors:** Khalid Nabil Nagshabandi, Abdulrahman M. Shadid, Abdulah Abdulsalam Almazro, Asem Shadid, Suad Shadid, Lamia Alakrash

**Affiliations:** ^1^ Department of Dermatology, College of Medicine, King Saud University, Riyadh, Saudi Arabia, ksu.edu.sa; ^2^ Department of Internal Medicine, Dr. Sulaiman Al-Habib Medical Group, Riyadh, Saudi Arabia; ^3^ Department of Dermatology, King Fahad Medical City, Riyadh, Saudi Arabia, kfmc.med.sa; ^4^ Department of Social Services, King Abdulaziz Medical City, Jeddah, Saudi Arabia, ngha.med.sa

**Keywords:** bariatric surgery, gastric bypass, keto rash, ketogenic diet, prurigo pigmentosa, review, Roux-en-Y gastric bypass, sleeve gastrectomy

## Abstract

Prurigo pigmentosa (PP) is a rare inflammatory dermatosis first described in 1971 by Nagashima, classically predominantly affecting young women, particularly those of East Asian descent. Clinically, PP presents with pruritic, erythematous papules, which eventually form a reticulated pattern and resolve into post‐inflammatory hyperpigmentation. The exact pathogenesis of PP remains unclear, but it is frequently linked to ketosis‐inducing conditions, including strict dieting, fasting, and metabolic changes, such as those observed in diabetic ketoacidosis or anorexia nervosa. In recent years, PP has been increasingly reported in patients undergoing bariatric surgery, likely due to the rapid weight loss and subsequent ketosis that often follow these procedures. This review aims to take a closer and deeper look at the emerging connection between PP and bariatric surgery, particularly laparoscopic sleeve gastrectomy.

## 1. Introduction

Prurigo pigmentosa (PP), also termed as Nagashima’s disease, or “keto rash”, is a rare inflammatory skin condition characterized by distinct clinical and histologic stages. PP was first described in 1971 by a Japanese dermatologist named Masaharu Nagashima and has since been recognized worldwide, although it remains rare outside East Asia [1]. The condition classically predominantly affects young women, particularly those of East Asian descent, and its etiology remains elusive, although various triggering factors have been identified [1]. Initially, it clinically manifests by pruritic urticarial papules, which then progress into crusted erythematous papulovesicles before it finally resolves into pigmented macules. Interestingly, different stages of the disease can coexist, creating a reticular pattern of post‐inflammatory hyperpigmentation primarily on the back, chest, and neck [1–3]. The histopathological features of PP change as the lesions progress through different stages. In the early stage, the histology shows a superficial perivascular neutrophilic infiltrate, with mild spongiosis and occasional necrotic keratinocytes, along with papillary edema in the dermis. As the lesions develop further, the fully developed stage is characterized by moderate spongiosis, a patchy lichenoid lymphocytic infiltrate, and numerous necrotic keratinocytes. Erythrocyte extravasation and hyperkeratosis are also common findings at this stage. In the late stage, inflammation subsides, and the histology primarily reveals melanophages in the papillary dermis, sparse lymphocytic infiltrates, hyperkeratosis with parakeratosis, and scale crusts. Pigment incontinence leads to the formation of pigmented macules, which are the hallmark of late‐stage lesions [1, 2]. A fully developed PP lesion under dermoscopy reveals several characteristic features. These include the presence of whitish scales, brown‐red structureless areas with indistinct borders, and multiple irregularly distributed brownish‐blue‐gray dots and globules. Additionally, blue‐white veil‐like structures can be observed over a background of erythema [5]. Many cases have linked PP to systemic diseases such as adult‐onset Still’s disease, Sjögren’s syndrome, atopy, and *Helicobacter pylori* infection [1]. The precise pathogenesis of PP remains unclear; however, it has been associated with various triggers such as ketosis, dieting, and metabolic changes, particularly in individuals following ketogenic or low‐carbohydrate diets. Other contributing factors include diabetes mellitus, diabetic ketoacidosis, physical stress, fasting, and restrictive diets (ketogenic) which may induce the metabolic alterations linked to PP. In addition to metabolic triggers, PP has been rarely associated with systemic inflammatory and autoimmune conditions (e.g., adult‐onset Still’s disease) [1, 4].

One emerging association in recent literature is PP developing in patients following bariatric surgery, particularly in the context of rapid weight loss and ketosis [6–13]. This clinicopathological review provides new insights into PP, particularly its intriguing association with bariatric surgery. We examine the clinical presentation and pathological basis of this condition, with a specific focus on its occurrence in patients who have undergone bariatric procedures. The aim of this review is to illuminate the potential relationship between these two entities and contribute to a more comprehensive understanding of PP.

## 2. Methodology and Materials

A comprehensive and systematic search of the literature was conducted using two major electronic databases: MEDLINE, accessed via PubMed, and Google Scholar. The search was performed in August 2024 with the goal of identifying relevant studies and case reports pertaining to PP in the context of metabolic changes following bariatric surgery. Specific search terms utilized in this review included “bariatric surgery,” “gastric bypass,” “ketogenic diet,” “keto rash,” “prurigo pigmentosa” “Roux‐en‐Y gastric bypass,” and “sleeve gastrectomy.” These search terms were carefully selected to capture both the metabolic triggers known to induce PP, such as the ketogenic state, and the specific surgical interventions like gastric bypass and sleeve gastrectomy. To ensure the comprehensiveness of the review, no language restrictions were applied, and all relevant publications up to August 2024 were considered. The search strategy was designed to identify both case reports and retrospective reviews that detailed the onset of PP following bariatric procedures, with a particular focus on how the metabolic changes induced by these surgeries may trigger the condition. After compiling an initial list of studies, duplicates were removed, and a thorough screening process was carried out based on predefined inclusion and exclusion criteria. Studies that lacked sufficient clinical detail, were not related to bariatric surgery, or did not involve a diagnosis of PP were excluded from the review.

## 3. Results

Following the application of the inclusion criteria, a total of eight documented cases of PP developing after bariatric surgery were identified in the existing literature. These cases spanned multiple geographic regions and encompassed a range of surgical techniques, including gastric bypass and sleeve gastrectomy. The findings are presented in chronological order, from the earliest published case to the most recent. This approach provides a clear overview of the progression in the recognition and reporting of PP as a potential complication of bariatric surgery, allowing for a better understanding of its clinical manifestations in this context. Each case was carefully analyzed, with a focus on the clinical presentation, patient demographics, type of bariatric surgery performed, time to onset of PP following the surgery, and the management strategies employed. A detailed summary of the clinical data, including symptoms, diagnostic methods, and treatment outcomes, from each case is provided in Table 1 [6–13]. This tabular presentation facilitates the comparison of key clinical features across the cases, enabling readers to appreciate patterns in the presentation and management of PP in this patient population.

**Table 1:** Summary of clinicopathological data from reported cases of prurigo pigmentosa following bariatric surgery.

Case (reference number) (region)	Year	Sex	Age (years)	Comorbid conditions	Type of bariatric surgery	Clinical presentation/site(s) affected	Onset	Histopathological characteristics	Management	Outcomes
Abbass et al. [6] (Lebanon)	2015	Female	40	NA	Laparoscopic sleeve gastrectomy	Symmetrically distributed erythematous papulovesicles and hyperpigmented macules arranged in a reticular pattern/chest and back	1 week post‐operation	Focal parakeratosis, mild epidermal hyperplasia, mild focal spongiosis, and a mild to moderately dense superficial and mid‐dermal perivascular and interstitial focally band‐like lympho‐neutrophilic infiltrate with scattered eosinophils	Oral doxycycline (100 mg, twice daily) and balanced diet	The eruption had not recurred at a 6‐month follow‐up post‐op

Al‐Dawsari et al. [7] (Saudi Arabia)	2019	Female	37	NA	Laparoscopic sleeve gastrectomy (complicated by splenic infarction and gastric leak development)	Multiple reticulated erythematous plaques and reticulated hyperpigmented patches/mid back, and mid chest Scattered urticarial plaques/right lateral back	12 days post‐operation	The epidermis showed focal parakeratosis and minimal spongiosis. Superficial and mid dermal perivascular cuffing by lymphocytes with rare eosinophils There was no interstitial inflammation	The patient was initially treated by the emergency services with intravenous and oral steroids with no response Was started on oral minocycline, but could not tolerate the related epigastric pain and stopped after 3 days of therapy	The rash resolved spontaneously at a 3‐month follow‐up post‐op

Alshaya et al. [8] (Saudi Arabia)	2019	Female	24	Prediabetic (on metformin)	Not mentioned	Scattered erythematous papules/sides of the face, shoulders, and chest	5 weeks post‐operation	Patient refused biopsy	Oral doxycycline 100 mg/d for 4 weeks	No recurrence of rash at a 2‐month follow‐up post‐op

Alsebayel et al. [9] (Saudi Arabia)	2020	Female	25	NA	Laparoscopic sleeve gastrectomy	Multiple erythematous reticulated papules and plaques with hyperpigmented macules/face, chest, and upper back/shoulder	3 days post‐operation	Mild vacuolar basal cell changes with focal lymphocytic exocytosis and scale crusting. Moderate superficial perivascular lymphocytic infiltrate with few neutrophils, eosinophils, and extraverted red blood cells	Oral minocycline extended‐release tablets (80 mg, QD for one month) Topical mometasone furoate cream (BID for one week)	Complete resolution of the eruption, with post‐inflammatory hyperpigmentation at a 1‐month follow‐up

Almuhaimeed et al. [10] (Saudi Arabia)	2021	Female	22	NA	Not mentioned	Erythematous papules and vesicles and papules‐vesicles/neck, chest, and upper back	4‐5 days post‐operation	Not done	Topical mometasone furoate cream (BID for one week)	Two‐week follow‐up showed improvement of the eruption

Alkhouri et al. [11] (United States of America)	2022	Female	25	NA	Laparoscopic sleeve gastrectomy	Small erythematous papules that progressed to becoming coalescent plaques with occasional crusted vesicles/chest.	3 weeks post‐operation.	Not done	High‐carb diet (stop ketogenic diet) Oral minocycline	After a three‐week follow‐up, the patient’s rash improved. Occasionally reappears one year post‐surgery during dieting.
Jazzar et al. [12] (Saudi Arabia)	2023	Male	25	Inflammatory bowel syndrome (taking gabapentin)	Not mentioned	Multiple erythematous papulovesicles in a reticular configuration/trunk, upper abdomen, and chest	18 days post‐operation	Focal interface reaction and scattered necrotic keratinocytes. Hair follicles were dilated and filled with bacteria. Mildly acanthotic dermis with perivascular lymphocytes, eosinophils, and extravasated red blood cells were also noted in the superficial dermis. There was no evidence of intraepidermal neutrophilic infiltrate	Topical clobetasol propionate ointment Oral minocycline 80 mg	At a two‐week follow‐up, the patient showed complete resolution of the rash and pruritus with residual post‐inflammatory hyperpigmentation in a reticular pattern

Alenizi et al. [13] (Saudi Arabia)	2023	Male	15	NA	Laparoscopic sleeve gastrectomy	Reticulated erythematous and hyperpigmented excoriated maculopapular rash/chest and back	3 weeks post‐operation	Patient refused biopsy	Oral doxycycline 100 mg daily for 4 weeks	At a 6‐week follow‐up, the lesions had healed with residual hyperpigmentation

*Note:* BID, two times a day; mg, milligram(s); QD, every day.

Abbreviation: NA = nonapplicable.

## 4. Discussion

PP, also referred to as Nagashima disease or “keto rash,” is an uncommon, inflammatory dermatosis that was first described by Nagashima et al. in 1971 [1, 2]. It classically affects young females, particularly those of East Asian origin, featuring a symmetric eruption of urticarial papules over the neck, chest, and back. The papules may coalesce into a reticulated pattern that frequently resolves and recurs, leading to hyperpigmentation that is often of cosmetic concern [2–4]. The cause of PP remains poorly understood, though it is thought to be an activation of the disorder by metabolic disorders like diabetes mellitus, dietary changes, hormonal fluctuations, or infections. Rare nonmetabolic associations have also been reported (e.g., adult‐onset Still’s disease) [1, 14, 15]. A relationship has been established between PP and metabolic states, such as ketoacidosis, fasting, and carbohydrate restriction—notably with some ketogenic diets. During PP exacerbations, levels of serum ketones higher than 0.6 mmol/L have been described, and the symptoms improved with normalization of urinary ketones [16]. PP has been increasingly recognized as a condition that can develop after bariatric surgery. The primary pathophysiological mechanism linking PP to bariatric surgery is thought to be related to ketoacidosis, a state commonly seen in patients undergoing significant weight loss or adopting restrictive dietary regimens. After bariatric surgery, rapid and drastic reductions in caloric intake can lead to the mobilization of fat stores and the production of ketone bodies as an alternative energy source. These elevated ketone levels are believed to trigger neutrophilic inflammation around blood vessels, leading to the hallmark erythematous macules, papules, and plaques seen in PP [17, 18]. This inflammatory response may be further aggravated by metabolic stressors such as nutrient deficiencies, which are common post‐surgery due to malabsorption [19]. The association of ketosis with PP has been proved in many instances related to fasting, insulin‐dependent diabetes, and anorexia nervosa [20–23]. Several case reports have documented the onset of PP in post‐bariatric patients, underscoring the importance of monitoring for this dermatological manifestation as a potential complication of the surgery.

A summary of the clinicopathological features from reported cases of PP following bariatric surgery is provided in Table 1 [6–13]. All cases reported were of young to middle‐aged adults, with ages ranging from 15–40 years. The majority of the patients were female (6 out of 8 cases), with only two male patients. Some patients had notable comorbid conditions. For example, one female patient was prediabetic and on metformin, and another male patient had inflammatory bowel syndrome and was taking gabapentin [8, 12]. For the remaining cases, comorbidities were either not mentioned or the patients did not have any known significant comorbidities. In all cases, patients were reported to undergo laparoscopic sleeve gastrectomy bariatric procedure. One case mentioned complications during surgery, including splenic infarction and gastric leak development [7]. The clinical presentations across all cases were consistent with erythematous papules, papulovesicles, and plaques arranged in reticulated patterns. The affected sites commonly included the chest, back, and shoulders, with a few cases affecting the face and neck. Time of onset varied, with skin lesions appearing 3–5 days post‐operation in some cases, while in others, onset occurred later, up to 5 weeks post‐surgery. Some patients did not undergo a skin biopsy [8, 10, 11, 13] while in other cases, the biopsies revealed typical findings consistent with the diagnosis of PP. Antibiotics such as doxycycline and minocycline were the most commonly prescribed treatments, typically administered for 3‐4 weeks. Some patients also received topical corticosteroids, such as mometasone furoate or clobetasol propionate ointment. One case noted the patient was treated with a high‐carb diet, suggesting dietary modifications as a management strategy to avoid ketogenic states, which may be a trigger for PP [11]. The outcomes were generally favorable. Most patients experienced complete resolution of their rash within a few weeks to months after treatment, though some cases showed post‐inflammatory hyperpigmentation as a residual effect. Notably, although PP has classically been described in East Asian populations, the majority of post‐bariatric PP cases identified in this review were reported from the Middle East, particularly Saudi Arabia (6 out of 8 cases), with additional cases from Lebanon and the United States (in a patient of Middle Eastern descent) [11]. This geographic clustering may reflect regional practice patterns, including the high and increasing volume of bariatric surgery in the Gulf/MENA region as well as reporting bias [37, 38]. Whether genetic, environmental, or cultural factors contribute to susceptibility in Middle Eastern populations remains uncertain and warrants further study.

The skin manifestations following bariatric surgery are multifaceted, often representing a mixture of improvements in certain conditions and the emergence of new challenges. Obesity‐related skin conditions, such as acanthosis nigricans, keratosis pilaris, and skin tags (acrochordons), tend to improve with successful weight loss post‐surgery. These improvements are primarily due to the amelioration of insulin resistance and metabolic dysregulation that are commonly associated with obesity. In contrast, some adverse effects, such as alopecia, may increase post‐surgery due to abrupt and significant weight loss, compounded by potential deficiencies in essential nutrients like zinc, iron, and vitamin B12. Additionally, post‐bariatric patients may develop skin laxity, striae distensae, and chronic venous insufficiency as a consequence of rapid weight loss and loss of structural skin integrity [24]. Although bariatric surgery has grown incredibly in popularity, only a few cases of PP have been reported to occur postoperatively [6–13]. Such underreporting may be a result of failure in recognizing and diagnosing the condition and/or may simply reflect the intrinsic rarity of the disease. Therefore, post‐surgical follow‐up often includes nutritional support to mitigate such adverse outcomes, in addition to meticulous documentation of cutaneous complications occurring postoperatively.

Given the high and increasing volume of bariatric surgery in Saudi Arabia and other Middle Eastern countries [37, 38], it becomes increasingly important to document and monitor PP as a potential postoperative complication. As bariatric surgery gains popularity as an effective treatment for morbid obesity, the incidence of ketoacidosis‐related conditions, such as PP, may rise. The rapid weight loss and metabolic shifts seen post‐surgery, particularly the induction of ketosis, are prime factors that can trigger PP [17, 18]. A study reported that the prevalence of obesity in Saudi Arabia is higher compared to the international average (35% vs. 13%) [25]. This high burden of obesity likely contributes to the increasing demand for bariatric surgery in the region. Given the growing number of surgeries performed annually in these regions, healthcare professionals should maintain a high index of suspicion for PP in postoperative patients presenting with pruritic, erythematous lesions [37, 38]. Documentation of cases, as well as raising awareness of this condition, is crucial for early diagnosis and intervention. Recommendations include thorough post‐surgical follow‐up, early nutritional supplementation to prevent extreme ketosis, and education for both patients and physicians about the signs and symptoms of PP to ensure timely and effective management.

The therapeutic options for managing PP usually include oral tetracycline and macrolide antibiotics. Oral minocycline (100 mg twice daily for 2–4 weeks) is considered the recommended first‐line therapy [26]. Tetracyclines act by suppressing inflammatory cytokines and neutrophil chemotaxis [27–30]. Macrolides and dapsone represent alternative regimens, the latter being a drug with well‐known effects on the inhibition of neutrophil chemotaxis [27, 31]. Adjunctive topical corticosteroids or calcineurin inhibitors have been applied to manage pruritus [32]. Treatment of persistent/post‐inflammatory hyperpigmentation is challenging, but some modalities have had success with laser therapy, chemical peels, and topical agents such as retinoids [33–35]. Strict sun protection is also advised to prevent the worsening of existing pigmentation [36].

## 5. Conclusion

PP is an underrecognized dermatological complication that may arise following bariatric surgery, particularly in the context of rapid weight loss and ketosis. As the prevalence of morbid obesity rates eligible for undergoing bariatric surgery continues to grow, heightened awareness and documentation of post‐op PP are essential for improving patient outcomes. Further research is warranted to explore potential genetic and environmental factors contributing to the increased susceptibility observed in certain populations.

## Ethics Statement

Ethical approval is not required for this study in accordance with local or national guidelines.

## Conflicts of Interest

The authors declare no conflicts of interest.

## Author Contributions

Khalid Nabil Nagshabandi, Asem Shadid, and Lamia Alakrash supervised the study. Khalid Nabil Nagshabandi, Asem Shadid, and Lamia Alakrash designed the study. Khalid Nabil Nagshabandi, Asem Shadid, Abdulah Abdulsalam Almazro, and Suad Shadid collected and analyzed the data. Khalid Nabil Nagshabandi, Asem Shadid, Abdulrahman M. Shadid, Abdulah Abdulsalam Almazro, Suad Shadid, and Lamia Alakrash participated in writing the manuscript. Khalid Nabil Nagshabandi, Asem Shadid, and Lamia Alakrash read and approved the final manuscript.

## Funding

No funding was received for this research.

## Data Availability

The data that support the findings of this study are available from the corresponding author upon reasonable request.
